# Sex Disparities in the Self-Evaluation of Subthalamic Deep Brain Stimulation Effects on Mood and Personality in Parkinson's Disease Patients

**DOI:** 10.3389/fneur.2020.00776

**Published:** 2020-07-31

**Authors:** Amelie D. Dietrich, Johannes A. Koeppen, Carsten Buhmann, Monika Pötter-Nerger, Hans O. Pinnschmidt, Christian Oehlwein, Marita Oehlwein, Katrin Mittmann, Christian Gerloff, Andreas K. Engel, Manfred Westphal, Miriam Schaper, Wolfgang Hamel, Christian K. E. Moll, Alessandro Gulberti

**Affiliations:** ^1^Department of Neurosurgery, University Medical Center Hamburg-Eppendorf, Hamburg, Germany; ^2^Department of Neurology, University Medical Center Hamburg-Eppendorf, Hamburg, Germany; ^3^Department of Medical Biometry and Epidemiology, University Medical Center Hamburg-Eppendorf, Hamburg, Germany; ^4^Neurological Outpatient Clinic for Parkinson's Disease and Deep Brain Stimulation, Gera, Germany; ^5^Department of Neurophysiology and Pathophysiology, University Medical Center Hamburg-Eppendorf, Hamburg, Germany

**Keywords:** Parkinson's disease, deep brain stimulation, subthalamic nucleus, personality traits, depression, non-motor symptoms, quality of life, sex differences

## Abstract

Changes in personality are one of the main concerns Parkinson's disease (PD) patients raise when facing the decision to undergo neurosurgery for deep brain stimulation (DBS) of the subthalamic nucleus (STN). While clinical instruments for monitoring functional changes following DBS surgery are well-established in the daily therapeutic routine, personality issues are far less systematically encompassed. Moreover, while sex disparities in the outcomes of STN-DBS therapy have been reported, little is known about the different effects that DBS treatment may have on mood and personality traits in female and male patients. To this aim, the effect of STN-DBS on personality traits was assessed in 46 PD patients (12 women and 34 men) by means of the Freiburg Personality Inventory. The Becks Depression Inventory (BDI-I) and the Parkinson's Disease Questionnaire were used to evaluate patients' level of depression and quality of life (QoL). Patients completed the questionnaires a few days before, within the first year, and 2 years after surgery. The 12 personality traits defined by the FPI-R questionnaire did not change significantly after STN-DBS surgery (*p* = 0.198). Women declared higher depression scores through all study stages (*p* = 0.009), but also showed a stronger QoL amelioration after surgery than male patients (*p* = 0.022). The BDI-I scores of female patients clearly correlated with their levodopa equivalent daily dose (LEDD; *r* = 0.621, *p* = 0.008). Remarkably, in both male and female patients, higher pre-operative LEDDs were related to worse post-operative QoL scores (*p* = 0.034). These results mitigate the concerns about systematic personality changes due to STN-DBS treatment in PD patients and encourage an early DBS approach, before severe levodopa-induced sequelae may irreparably compromise the patients' QoL. In the future, more focus should lie on sex-related effects, since female patients seem to profit more than male patients from STN-DBS, in terms of reduced depressive symptoms associated with a reduction of the LEDD and amelioration of QoL. These aspects may help to redress the sex imbalance in PD patients treated with DBS, given that women are still strongly under-represented.

## Introduction

For selected patients with Parkinson's disease (PD), neurosurgery for deep brain stimulation of the subthalamic nucleus (STN-DBS) is a proven therapy that simultaneously improves motor function and allows a significant reduction of dopaminergic medication (1–3). Numerous scientific reports on the long-term effects of STN-DBS demonstrate the link between clinical improvements in motor symptoms and improvements in health-related quality of life [QoL; (4–11)]. Nevertheless, potential adverse effects of STN-DBS on behavior, mood, and personality are among the main concerns patients and caregivers voice when deciding whether or not to undergo neurosurgery (12–18). In fact, changes in mood and psychosocial functions in PD patients after surgery for STN-DBS have been reported previously (19–24). Other studies have shown that adverse psychiatric symptoms, like depression, hallucinations, and confusion, have a long-term negative effect on the QoL of patients treated with STN-DBS. However, these studies are limited by the lack of reliable instruments for assessing patients' reflection of personality and emotional-state changes (18, 25, 26). Furthermore, while sex differences in the clinical characteristics of PD and in the outcomes of STN-DBS therapy have been reported, little is known about the distinct effects that DBS treatment may have on mood and personality traits in female and male patients (27–32). Facing these challenges with the PD patients operated on in our institution, we intended to keep track of the long-term effects of chronic STN stimulation on mood and personality, and their influence on the patients' QoL, focusing in particular on possible sex disparities. In search of a valid instrument for capturing the subtle personality alterations that may appear in patients undergoing STN-DBS, we chose the Freiburg Personality Inventory (FPI-R), a widely used and accepted personality inventory in the German language (33–36). Patient-reported outcomes were specifically chosen, since a discrepancy between the effects of the STN-DBS therapy as estimated by the patients and by the clinicians can be critical for the success of the therapy itself (37, 38).

## Materials and Methods

### Design

This was a single-center, longitudinal study to determine possible changes in personality, mood and QoL of female and male PD patients undergoing neurosurgery for STN-DBS. The study was conducted as a prospective exploratory pilot trial. To avoid selection bias, all of the 46 PD patients treated with STN-DBS therapy at our institution during the investigation period were included. They were asked to fill out a series of standardized questionnaires (see below) at three planned stages: the week before surgery (pre-operation), between 3 and 12 months after surgery (1st post-operation; average follow-up time: 9 months), and between 15 and 36 months after surgery (2nd post-operation; average follow-up time: 26 months; see Table 1 for details).

**Table 1:** Clinical and demographic characteristics at the date of DBS neurosurgery, unless noted otherwise.

									**Pre-op UPDRS**		**1st post-op DBS parameters**			**1st post-op UPDRS**			**2nd post-op DBS parameters**			**2nd post-op UPDRS**	
**Case** **Sex** **Age**			**Disease duration (years)**	**Pre-** ** op**	**1st** ** post**	**2nd** ** post**	**H&Y**	**Pre-op** ** LEDD mg**	**DOPA-OFF**	**DOPA-ON**	**Left electrode (row 1) ** ** Right electrode (row 2)**	**1st post-op LEDD mg**	**H&Y**	**DOPA-OFF OFF-DBS**	**DOPA-OFF ON-DBS**	**DOPA-ON ON-DBS**	**Left electrode (row 1)** ** Right electrode (row 2)**	**2nd post-op LEDD mg**	**H&Y**	**DOPA-OFF OFF-DBS**	**DOPA-OFF** ** ON-DBS**	**DOPA-ON** ** ON-DBS**
2	F	58	3	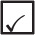	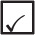	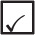	1	1543.8	39	6	130 Hz, 2–, 2 V, 60 μs 130 Hz, 10–, 1.9 V, 60 μs	860.25	2	45	34	22	130 Hz, 2–, 2 V, 60 μs 130 Hz, 9+10–, 3.2 V, 90 μs	1064				
3	F	60	15	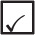	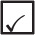	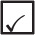	3	669.06	27	14	130 Hz, 2–, 2.4 V, 60 μs 130 Hz, 10–, 2.4 V, 60 μs	1210	2				130 Hz, 2–, 2.5 V, 60 μs 130 Hz, 10–, 2.4 V, 60 μs	537.5	2			
21	F	57	13			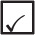		1147	49	9	130 Hz, 1–, 1.6 V, 60 μs 130 Hz, 10–, 2.6 V, 60 μs	758.75	5	49	24	12	180 Hz, 2–, 2.2 V, 60 μs 180 Hz, 10–, 3.2 V, 90 μs	607		32	11	9
29	F	70	14	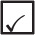	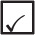	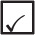		475	17	20	180 Hz, 2–, 2.5 V, 90 μs 180 Hz, 11–, 3 V, 60 μs	498.75	3			13	130 Hz, 1–2–, 1.7 V, 90 μs 130 Hz, 9–10–, 1.5 V, 90 μs	385	3			20
30	F	54	9	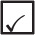	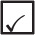	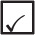	3	2831.4	39	24	130 Hz, 1–3–, 1.9 V, 60 μs 130 Hz, 11–, 3.3 V, 60 μs	840	3			29	130 Hz, 2–3–, 2.9 V, 60 μs 130 Hz, 8–9–11+, 3.2 V, 90 μs	1356.9	4			19
31	F	68	13	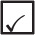	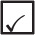	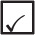		787.5	31	17	130 Hz, 1–2–, 2.5 V, 60 μs 130 Hz, 9–10–, 3.4 V, 60 μs	994.38	3			7	180 Hz, 1–2–, 2.1 V, 60 μs 180 Hz, 9–10–, 2.9 V, 90 μs	1092.8				
32	F	55	13	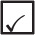	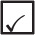	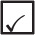	2	1287.5	23	10	130 Hz, 2–, 1.3 V, 60 μs 130 Hz, 10–, 2.3 V, 60 μs	515.62	1.5			7	210 Hz, 1–2–, 2 V, 60 μs 210 Hz, 9–10–, 2.5 V, 60 μs	1264.4	1.5			5
40	F	56	9	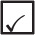	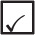	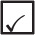	3	1981.2	67	10	130 Hz, 2–3–, 2.2 V, 60 μs 130 Hz, 10–11–, 2.5 V, 60 μs	912.5		53	22	17	130 Hz, 1+2–, 2.7 V, 60 μs 130 Hz, 9+10–, 2.9 V, 60 μs	1250	3			
44	F	61	15	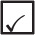	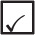	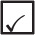	3	1201.6	47	11	130 Hz, 1–2–, 2.6 V, 60 μs 130 Hz, 9–10–, 2.6 V, 60 μs	799.38	3	39	27	29	120 Hz, 1–, 3.9 V, 60 μs 120 Hz, 9–, 3.5 V, 60 μs	1073.2	3			
46	F	44	7	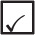	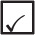	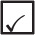	2	835	46	15	180 Hz, 1–2–, 5 V, 60 μs 180 Hz, 10–, 2.8 V, 60 μs	612.25	2			3	180 Hz, 1–2–, 5 V, 60 μs 180 Hz, 10–, 2.8 V, 60 μs	764	2			
48	F	71	12	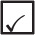	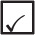	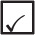	3	367.62	20	9	185 Hz, 2–, 1.5 V, 60 μs 185 Hz, 10–11–, 4 V, 60 μs	332.62					185 Hz, 2–, 1.5 V, 60 μs 185 Hz, 10–11–, 3.5 V, 60 μs	332.62	3			
49	F	70	6	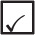	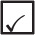	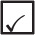		1061.4	28	7	130 Hz, 2–, 1.8 V, 60 μs 130 Hz, 10–, 2.2 V, 60 μs	682										
***Women***	**12 F**	**60.0.3**	**10.8**				**3**	**1182.34**	**35**	**10.5**		**751.4 (−36.4%)**	**3**	**47**	**25.5**	**13**		**884.3 (−25.2%)**	**3**	**32**	**11**	**14**
1	M	66	27	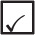	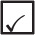	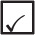	4	2554.7	30	14	130 Hz, 2–, 2.3 V, 60 μs 130 Hz, 10–, 2 V, 60 μs	1115.2	3			9	140 Hz, 2+3–, 2.7 V, 60 μs 140 Hz, 10+11–, 3.4 V, 60 μs	812.81	4			
4	M	68	9		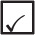			1080	24	16	130 Hz, 2–, 2.7 V, 60 μs 130 Hz, 9–, 2.7 V, 60 μs	351.75	2			1	130 Hz, 2–3–, 2.5 V, 90 μs 130 Hz, 9–10–, 2.5 V, 90 μs	400	1.5			7
5	M	71	24	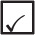	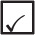	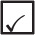	3	1161.9	47	32	130 Hz, 3–, 2.4 V, 60 μs 130 Hz, 10–, 2.8 V, 60 μs	816.88	2	46	41	37	130 Hz, 2–3–, 2.5 V, 60 μs 130 Hz, 9–10–, 2.7 V, 60 μs	816.88	3			15
6	M	57	11			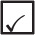	3	1414	54	10	130 Hz, 1–2–, 2.5 V, 60 μs 130 Hz, 9–10–, 2.5 V, 60 μs	2005.8	3	33	27	21	130 Hz, 1–2–, 3.2 V, 60 μs 130 Hz, 9–10–, 2.8 V, 60 μs	1280.2	4			
7	M	45	7	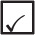		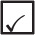		490.88	25	17	130 Hz, 2–, 3.4 V, 60 μs 130 Hz, 10–, 3.2 V, 60 μs	346.88	4	34		17	130 Hz, 1–2–, 3.1 V, 60 μs 130 Hz, 9–10–, 3 V, 60 μs	591.88	2			9
8	M	72	8	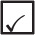	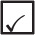	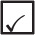	3	2551.9	47	23	130 Hz, 2–, 3.7 V, 60 μs 130 Hz, 9–10–, 3.4 V, 60 μs	1012.5	3			12	130 Hz, 2–, 3.7 V, 60 μs 130 Hz, 9–10–, 3.4 V, 60 μs	881.25	4			
9	M	45	7	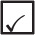			3	835.12	49	16	210 Hz, 1–, 2.5 V, 60 μs 210 Hz, 9–, 2.5 V, 60 μs	425.56	3	20		25	210 Hz, 1–, 2.5 V, 60 μs 210 Hz, 9–, 2.5 V, 60 μs	842.88	3			
10	M	71	14	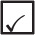		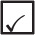		1614.1	39	31	130 Hz, 3–, 3 V, 60 μs 130 Hz, 11–, 3 V, 60 μs	1012.8	3	44	36	19	130 Hz, 3–, 3.2 V, 60 μs 130 Hz, 11–, 3.2 V, 60 μs	1114.1	3			20
11	M	54	9		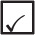	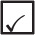		2052.5	32	16	130 Hz, 2–, 2.3 V, 60 μs 130 Hz, 10–, 3.1 V, 60 μs	658.44		31	25	23	125 Hz, 2–3–, 2.6 V, 60 μs 125 Hz, 10–, 3.6 V, 60 μs	1419.3	3			19
12	M	68	13	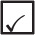	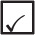	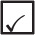	2	707		28	130 Hz, 2–, 3.1 V, 60 μs 130 Hz, 10–, 3.3 V, 60 μs	478.12	2			8	130 Hz, 2–, 2.4 V, 60 μs 130 Hz, 10–, 2.4 V, 60 μs	2899.4	2			28
13	M	70	9	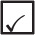		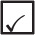	3	1589.9	36	14	130 Hz, 1–, 2.3 V, 60 μs 130 Hz, 10–, 2.8 V, 60 μs	1745.8	3	38	24	19	130 Hz, 1–2+, 1.8 V, 60 μs 130 Hz, 9–10+, 2 V, 60 μs	2066.9	3			
15	M	62	13	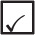	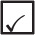	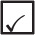	1	776.25	15	9	130 Hz, 1–, 2.1 V, 60 μs 130 Hz, 9–10–, 3.7 V, 60 μs	356.25			14	10	130 Hz, 1–, 1.5 V, 60 μs 130 Hz, 9–10–, 3.9 V, 60 μs	566.25				
16	M	60	17	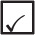	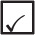	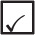		931.69	44	20	130 Hz, 1–2–, 2.5 V, 60 μs 130 Hz, 9–10–,3 V, 60 μs	910.5	3				120 Hz, 1–2–, 4 V, 60 μs 120 Hz, 9–10–, 2.4 V, 60 μs	2294.3	3			
17	M	69	19			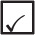	3	1365.6	29	11	130 Hz, 1–2+, 2.4 V, 60 μs 130 Hz, 9–, 3 V, 60 μs	958.75		32	25	9	130 Hz, 2–3+, 3 V, 60 μs 130 Hz, 9+10–, 3.8 V, 60 μs	998.75	3	41	29	21
18	M	59	9	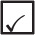		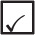	3	1692.2	46	14	130 Hz, 2–, 2 V, 60 μs 130 Hz, 9–, 2.6 V, 60 μs	1733	4			31	130 Hz, 2–, 2.1 V, 60 μs 130 Hz, 9–, 2.6 V, 60 μs	1733	4			
19	M	67	2		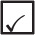	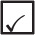	4	1431.9	39	23	130 Hz, 2–3–, 2.2 V, 60 μs 130 Hz, 9–10–, 3 V, 60 μs	661.88	2			11	130 Hz, 2–3–, 2.2 V, 60 μs 130 Hz, 9–10–, 3 V, 60 μs	816.88	4			11
20	M	63	13	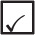	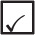	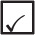		1214	36	12	130 Hz, 1–, 3 V, 60 μs 130 Hz, 9–, 2.8 V, 60 μs	350	3				130 Hz, 1–2–, 3 V, 60 μs 130 Hz, 9–10–, 2.8 V, 60 μs	445	2			10
22	M	57	13	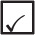	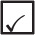			1135			180 Hz, 2–, 2 V, 60 μs 180 Hz, 11–, 1.5 V, 60 μs	866.25	3			25	180 Hz, 2–, 3.3 V, 60 μs 180 Hz, 11–, 3 V, 60 μs	1205	3			
23	M	68	14	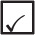			3	2275.6	31	17	130 Hz, 2+3–, 4.8 V, 60 μs 130 Hz, 9–, 3 V, 60 μs	1124.3	3		42	28	130 Hz, 2–3+, 4.8 V, 60 μs 130 Hz, 10–, 3 V, 60 μs	1138.1	4			
26	M	68	3	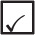		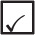	2	1541.2	31	9	130 Hz, 2–, 3 V, 60 μs 130 Hz, 10–, 3.4 V, 60 μs	1518.8	3				130 Hz, 3–, 2.8 V, 60 μs 130 Hz, 11–, 3.6 V, 60 μs	1350	4	42	30	13
27	M	70	7	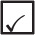	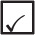	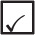	3	758.75	32	15	130 Hz, 2–, 3.1 V, 60 μs 130 Hz, 10–11+, 4 V, 60 μs	475	2			10	180 Hz, 3–, 2.6 V, 60 μs 180 Hz, 11–, 3.3 V, 60 μs	707.62	3			
28	M	61	11	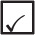		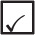	3	1819.1	51	44	160 Hz, 2–, 3.3 V, 60 μs 160 Hz, 10–, 3.5 V, 60 μs	505.56	3			19	70 Hz, 2–, 3.5 V, 60 μs 70 Hz, 10–, 3.7 V, 60 μs	1040.8				
33	M	73	12	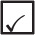		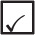	3	1792.5	45	10	130 Hz, 2–, 1.8 V, 60 μs 130 Hz, 10–, 2.2 V, 60 μs	157	3			2	140 Hz, 2–, 2.3 V, 60 μs 140 Hz, 10–, 3.5 V, 60 μs					
34	M	61	15	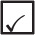			4	1072.2	35	13	130 Hz, 2–, 3.4 V, 60 μs 130 Hz, 10–, 4.2 V, 60 μs	397.5				24	130 Hz, 2–, 3.2 V, 60 μs 130 Hz, 9–10+, 4 V, 60 μs	713.75				
35	M	63	13	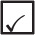		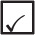	2	1239.1	28	22	130 Hz, 2–, 2.4 V, 60 μs 130 Hz, 10–, 2.4 V, 60 μs	400	1			5	210 Hz, 2–3–, 3.2 V, 90 μs 210 Hz, 10–11–, 3.1 V, 60 μs	457.81	2			11
36	M	40	6	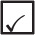	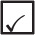	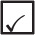	4	950	40	24	130 Hz, 1–2–, 1.6 V, 60 μs 130 Hz, 9–10–, 1.9 V, 60 μs	398.44	2	20	8	7	125 Hz, 2–, 3 V, 60 μs 125 Hz, 10–, 2.3 V, 60 μs	473.44	2			8
37	M	64	6	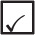	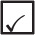	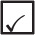	4	2034.4	32	21	130 Hz, 3–, 1.6 V, 60 μs 130 Hz, 10–, 2.4 V, 60 μs	1758.8	4			10	130 Hz, 2–3–, 3 V, 60 μs 130 Hz, 9–10–, 3 V, 60 μs	1731.9				
38	M	63	5	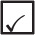	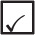		2.5	356.25	39	24	180 Hz, 2–, 4.3 V, 60 μs 180 Hz, 10–, 1.4 V, 60 μs	356.25	1	47	14	28	180 Hz, 2–, 4.1 V, 90 μs 180 Hz, 10–, 1.7 V, 60 μs	313.12	2.5			
39	M	69	12	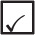	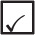		4	1593.8	63	28	130 Hz, 2–, 4.2 V, 60 μs 130 Hz, 10–, 4 V, 60 μs	1108.4	4	31	17	16	130 Hz, 2–, 2.2 V, 60 μs 130 Hz, 10–, 4 V, 60 μs	1292.5	4			
41	M	48	5			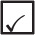	3	1986.4	26	7	130 Hz, 2–, 1.7 V, 60 μs 130 Hz, 10–, 3.2 V, 60 μs	707				15	125 Hz, 2–, 2.6 V, 60 μs 125 Hz, 10–, 3.2 V, 60 μs	1286.2	3			
42	M	66	6	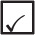		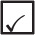	2.5	2251.2	48	22	130 Hz, 2–, 1.9 V, 60 μs 130 Hz, 10–, 0.6 V, 60 μs	776.25				7						
43	M	69	12	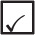	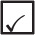	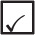		1973.8	17	10	130 Hz, 3–, 2.1 V, 60 μs 130 Hz, 10–, 2.1 V, 60 μs	777.5	1.5			14		1611.8	2			20
45	M	67	22	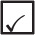	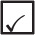	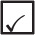	3.5	1491.2	51	27	130 Hz, 2–, 2.4 V, 60 μs 130 Hz, 10–, 2.5 V, 60 μs	782.62	3.5			6	130 Hz, 2–3–, 2.5 V, 60 μs 130 Hz, 10–11–, 3 V, 60 μs	818.44	3			18
47	M	69	4	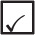	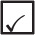	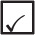	3	1233.8	30	26	130 Hz, 3–, 2.4 V, 60 μs 130 Hz, 11–, 1.7 V, 60 μs	545.31	3			12	210 Hz, 2–3–, 2.7 V, 90 μs 210 Hz, 10–11–, 3.1 V, 60 μs		3			
**Men**	**34 M**	**63.0**	**11.1**				**3**	**1440.23**	**36**	**17**		**811.62 (−43.6%)**	**3**	**32.5**	**25**	**13**		**1100.65 (−23.6%)**	**3**	**41.5**	**29.5**	**14**
**Summary**	**12 F & 34 M**	**62.3**	**11.0**				**3**	**1372.96**	**36**	**16**		**795.90 (−42.0%)**	**3**	**38.5**	**25**	**13**		**1043.99 (−24.0%)**	**3**	**41**	**29**	**14**

*In the “Disease duration (in years)” column, the disease duration is calculated from the date of the first diagnosis to the date of DBS surgery. In columns “Pre-op,” “1st post,” “2nd post,” the check boxes indicate the stages at which the patients answered the questionnaires. In the “DBS parameters” column, values reported are stimulation frequency in Hz, active contacts, impulse amplitude in volts, and impulse width in microseconds for the left and right electrode. The neurostimulator case is set as positive (anode) and the active contacts as negative (cathodes, contact number followed by a minus sign). In the case of a bipolar configuration, the most effective contact is set as negative and the adjacent contact as positive. For the left electrode, contact 0 was the most ventral and contact 3 was the most dorsal. For the right electrode, contact 8 was the most ventral and contact 11 was the most dorsal. H&Y, Hoehn & Yahr scale; Op, operation for DBS electrodes implantation; UPDRS-III, Unified Parkinson's Disease Rating Scale, motor-subscore (part III); LEDD, levodopa equivalent daily doses. In the “summary-rows” the mean values are reported for “years” and “LEDD mg,” while median values are reported for the Hoehn & Yahr and UPDRS-III scales; the mean post-operative LEDD reduction in comparison to the pre-operative baseline is given as %. Some of the clinical data were collected retrospectively, and not all clinical scores matching the date of the completion of the questionnaires could be successfully retrieved*.

### Participants

The survey was conducted on 46 consecutive PD patients undergoing bilateral implantation of DBS electrodes in the STN at the Department of Neurosurgery at the University Medical Center Hamburg-Eppendorf (12 women and 34 men, ranging in ages from 40 to 73; mean age: 62.3 ± 8.3 years). The uneven distribution of women and men reflects the worldwide gap between male and female PD patients undergoing neurosurgery for STN-DBS (39, 40). Mean disease duration was 11.0 ± 5.3 years with a median Hoehn & Yahr stage of 3.0 ± 0.4 IQR (41). On average, participants had attended school for 10.3 ± 1.7 years. Pre-operatively, all patients demonstrated an adequate global intellectual capacity, when tested with the Mini-Mental State Examination (42) (median score: 29 ± 1 IQR) and the DemTect (43) (median score: 15 ± 4 IQR). Furthermore, they fulfilled other inclusion criteria for STN-DBS, such as no structural alterations on magnetic resonance imaging and no severe medical comorbidities. All participants had to read and sign an informed consent before participating in the study. The study was approved by the local ethics committee and was conducted in agreement with the Code of Ethics of the World Medical Association (Declaration of Helsinki, 1967). Further clinical details are summarized in Table 1.

### Questionnaires and Outcome Measures

The Freiburg Personality Inventory Revised (FPI-R) is a structured verbal omnibus test, which is widespread in German-speaking countries and used for multidimensional measurement of normal personality traits (33, 35, 36). Some aspects of the FPI-R are similar to the Minnesota Multiphasic Personality Inventory (44) and more generally to the Eysenck's Personality Inventory (33, 45, 46) and to the Sixteen Personality Factor Questionnaire (47, 48). It is comprised of 138 statements compiled into 10 standard scales, which are more relevant for psychological research and diagnostics. Two additional scales, relating to the two basic factors “Extraversion” and “Emotionality,” as introduced by Eysenck, are also included (33, 49–51). The 12 scales reported are (1) “Life Satisfaction,” (2) “Social Orientation,” (3) “Performance Orientation,” (4) “Shyness,” (5) “Irritability,” (6) “Aggressiveness,” (7) “Stress,” (8) “Physical Complaints,” (9) “Health Concerns,” (10) “Openness,” (11) “Extraversion,” (12) “Emotionality.” Every statement must be answered by ticking “right” or “wrong.” For evaluation, the individual raw scores are subdivided into the 12 scales and summarized as a graphical profile with stanine values. High stanine values (from 1 to 9) indicate a strong expression of the respective personality trait.

The Becks Depression Inventory (BDI-I) is a 21-item questionnaire to assess depressive symptoms with a score of 0–3 for each item. A maximum of 63 points can be achieved (higher scores indicate worse symptoms). In parkinsonian populations, a score of 16 is considered a good cut-off for diagnostic purposes (52, 53).

The Parkinson's Disease Questionnaire is a 39-item self-reported questionnaire (PDQ-39) that records frequent and specific health-related problems over previous monthly periods and reliably reproduces the QoL of PD patients (54, 55).

### Statistics

To deal with missing data and unequal sample sizes, a mixed model approach was chosen (56). The scores were compared at the three experimental stages (pre-operation, 1st post-operation, 2nd post-operation) for sex (male vs. female patients; primary independent variables). The 12 FPI-R categories were also considered as primary independent variables. The primary endpoints were the scores of the 12 personality traits of the FPI-R, the level of depression as measured by the BDI-I, and the sum score “PDSI” of the PDQ-39. Secondary independent variables included the pre-operative Hoehn & Yahr (H&Y) stage, age at surgery, LEDD, and the pre-operative levodopa response, as an expression of the most common and accepted clinical scores collected in PD therapies. Prior to analysis, all continuous variables with a positively skewed distribution were natural log transformed to achieve normal distribution; negatively skewed distributions were first reverse-score transformed before the natural log transformation (57). The dependent variables were analyzed using a general linear mixed model approach with an identity-link function assuming normally distributed data (SPSS routine GENLINMIXED; IBM SPSS Statistics for Mac, version 25.0.0.2, SPSS Inc., Chicago, IL, USA). DBS time, sex, age at surgery, H&Y stage at surgery, and LEDD at surgery were considered as categorical fixed-effect variables, as well as their interaction terms with the primary independent variables. The patient was assumed as a random effect and the three planned stages as repeated measures within a patient (DBS time), applying a 1st order autoregressive (AR1) covariance structure for the residuals. The approximate degrees of freedom (df) were computed according to the Satterthwaite method and robust standard errors according to the Huber-White method. Starting from an initial model containing all fixed main effects of primary independent variables, their two-way interaction terms, and main effects of secondary independent variables, non-significant interaction terms, and secondary independent variables were stepwise excluded following a hierarchical backward elimination procedure based on maximum likelihood estimation (58). The final models contained all main effect terms of the primary independent variables and their significant interaction terms, as well as the significant main effects of the secondary independent variables and their significant interaction terms with the primary independent variables. Model computations were adjusted for baseline values of the respective dependent variables. The GENLINMIXED-estimated marginal means and their 95% confidence intervals (CIs) were computed for all dependent variables at all study stages, followed by *post-hoc* pairwise comparisons of the three study stages by means of linear contrasts; the sequential Sidak-adjusted significance level was 0.05. As this was an exploratory pilot study, no further adjustments for multiple testing were done (59). In case of significant two-way interaction effect terms, *post-hoc* repeated measures correlations were performed using the *rmcorr* R package [R version 3.5.0; *rmcorr* package by (60)]. This method was applied to assess consistencies between the self-evaluation scores and the clinical scores (i.e., LEDD, H&Y) as determined by the physicians at the three planned stages. By removing measured variance between participants, rmcorr provides the best linear fit for each participant using parallel regression lines (the same slope) with varying intercepts (60). Missing single items have been handled according to the official guidelines reported for the standardized questionnaires.

### Electrode Implantation and Localization of the Active Contacts

An exhaustive description of the microelectrode recording (MER) routine for mapping the STN, and of the surgical procedure for DBS lead implantation at our institution has been reported in previous works (61, 62). In short, individually adjusted target coordinates were planned fusing preoperatively acquired high-resolution T2-weighted magnetic resonance images with stereotactic computerized tomography (CT) scans. The dorsal STN was targeted 10.25–13 mm lateral to midline, 1–2 mm posterior to the mid-commissural point, and 2 mm inferior to the inter-commissural plane. The great majority of patients (43/46) were awake and co-operative during stereotactic DBS electrode placement. In only 3 patients, DBS surgery had to be carried out under general anesthesia (63). In all patients, final lead placement was adjusted following the information provided by the MERs and by the intraoperative test stimulations. Therefore, up to five parallel tracks (4 ± 1) arranged in a concentric configuration were used to map the STN area with tungsten microelectrodes (NeuroProbe electrodes and MicroGuide system, Alpha Omega Inc., Nazareth, Israel). The subthalamic sensorimotor region was identified by cell responses to passive and active movements and a high prevalence of oscillating neuronal activities in the beta-frequency range (13–30 Hz). A clear increase in the back-ground cell activity signaled the entrance of the micro-tips into the STN. The STN neurons were characterized by a tonic irregular, oscillatory bursting activity, clearly distinguished from the overlying thalamus/zona incerta neurons, displaying a slower bursting and single spiking activity, and from the underlying high-frequency regular spiking activity of substantia nigra pars reticulata neurons (61, 64). The reconstruction of the active DBS lead contacts (model 3389; Medtronic, Minneapolis, Minnesota, USA) was performed by co-registration of the preoperative T1 MRI scans and post-operative CT scans using iPlan (iPlan stereotaxy; Brainlab, Feldkirchen, Germany). Further details concerning the localization of active electrode contacts were reported previously (65, 66). In patients with two adjacent active contacts (cathodes), the averaged x, y, z stereotactic coordinates of those pairs were considered for statistical testing (see Table 1).

## Results

### The Freiburg Personality Inventory Revised (FPI-R)

**Figure 1:**
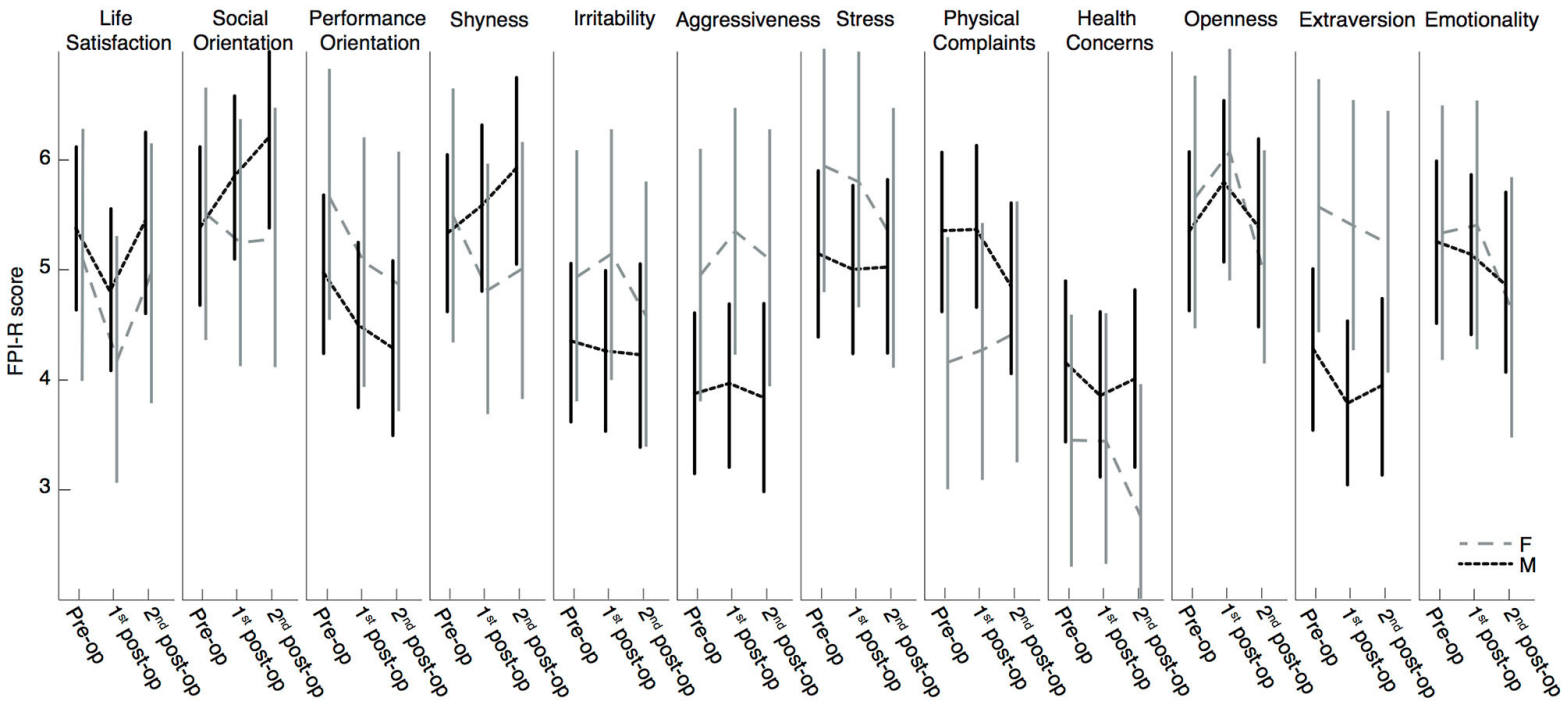
Estimated marginal means of FPI-R scores with 95% CI. Assessment of 10 subscales and two additional scales (Extraversion and Emotionality) to analyze the effect of DBS on the personality of the patient: pre-operation, 1st post-operation, 2nd post-operation separated by sex: F = female, M = male. High stanine values (from 1 to 9) indicate a strong expression of this personality trait.

The generalized linear mixed model revealed that the 12 categories of the FPI-R questionnaire were not significantly modulated by the factor DBS time: the personality traits of the 46 PD patients did not significantly change after STN-DBS surgery. The factor sex did not have any significant effect or interaction with the factors DBS time or FPI scales (see Table 2, Figure 1). Planned *post-hoc* pairwise contrasts indicated that patients reported a reduction in the category “Life Satisfaction” between pre-operation and the 1st post-operative stage (*p* = 0.012, Sidak corrected) and returned to pre-operative levels at the 2nd post-operative stage (*p* = 0.658, Sidak corrected; see Figure 1). In the category “Performance Orientation,” there was a significant score decrease between the pre-operative and the 2nd post-operative stage (*p* = 0.028, Sidak corrected; see Figure 1).

**Table 2:** Fixed effects of the generalized linear mixed model and post-hoc repeated measures correlations for the PDQ-39 sum score PDSI.

**Source**	***F***	**df1**	**df2**	***p*-values**
DBS-Time	1.624	2	650	0.198
Sex	0.230	1	43	0.634
DBS-time * FPI scales	1.235	22	689	0.210
FPI scales * Sex	1.444	11	445	0.150

The reported values for the fixed effects are degrees of freedom (df1 and df2), F and p-values.

### The Becks Depression Inventory (BDI-I)

**Figure 2:**
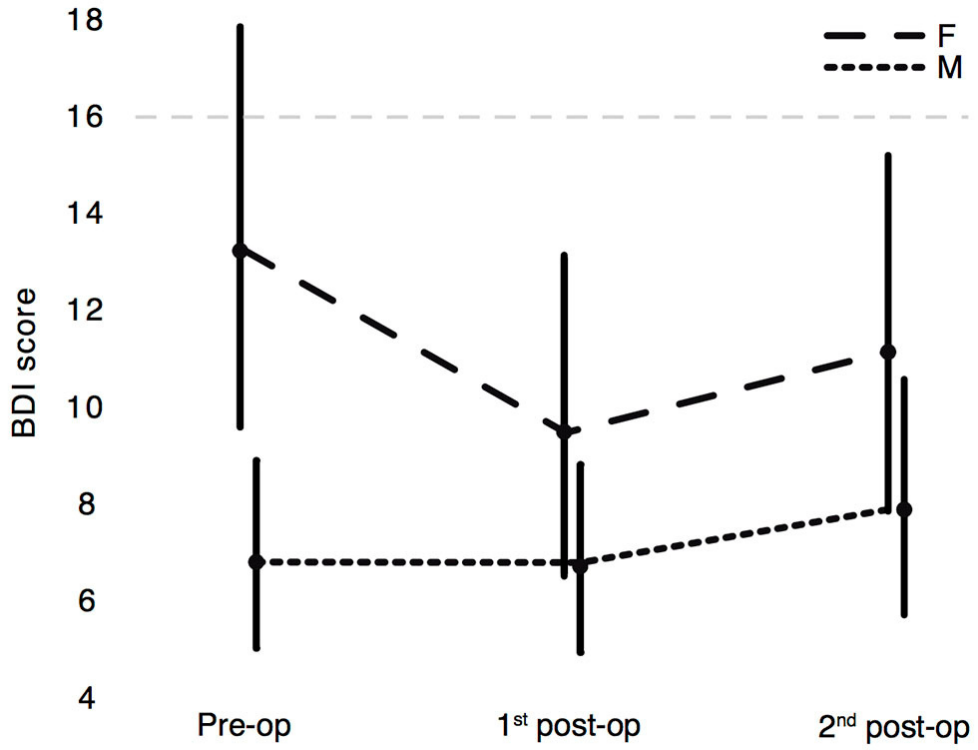
Estimated marginal means of BDI-I scores with 95% CI. Assessment of depressive symptoms in PD patients at the three experimental stages: pre-operation, 1st post-operation, 2nd post-operation separated by sex: F = female, M = male. The dashed horizontal line at value 16 represents the cut-off for PD patients.

On average, our cohort did not meet the criteria for depression at any of the three planned stages (pre-operation: 9.7 ± 7.1; 1st post-operation: 8.5 ± 5.7; 2nd post-operation: 9.9 ± 5.7; clinical cut-off score: 16). The general linear mixed model revealed that the BDI-I score was not significantly modulated by the factor DBS time, but it was modulated by sex: female patients had significantly higher BDI scores than male patients, independent of study stage (Figure 2).

**Figure 3:**
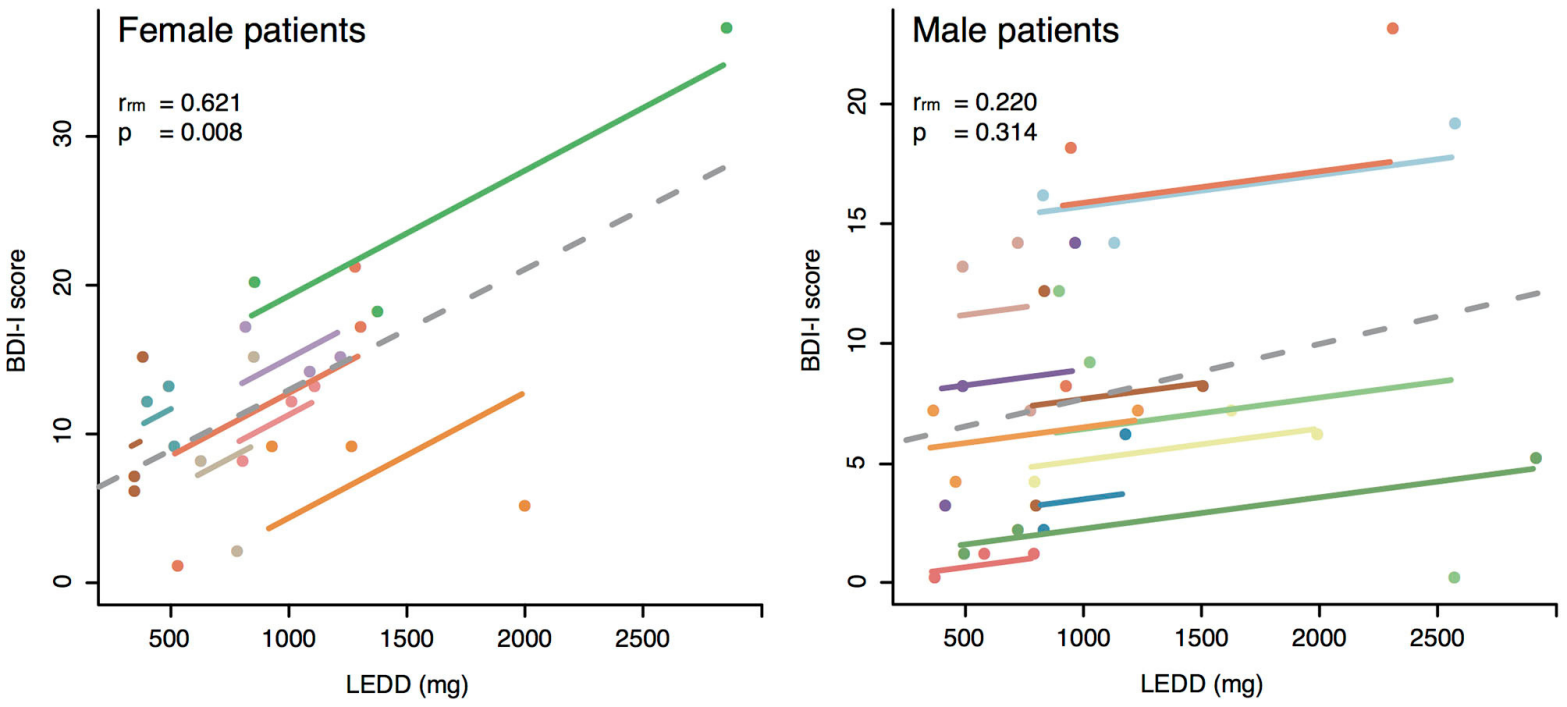
Repeated measures correlations. The left panel represents BDI-I scores vs. LEDD (mg) for female PD patients. The right panel represents BDI-I scores vs. LEDD (mg) for male PD patients. Color coded are the single patients at the three planned stages. The separate parallel lines show the rmcorr fit for each individual patient. The sign of the rmcorr coefficient (i.e., positive or negative) is indicated by the direction of the common regression slope plotted as dotted line. Inset values give the statistics for the corresponding repeated measures correlation.

Whereas *post-hoc* repeated measures correlations performed for the three planned stages did not show any significant correspondence between the H&Y and BDI-I scores for either female or male patients, a clear correlation was found between LEDDs and BDI-I scores of female patients (*p* = 0.008) but not for male patients (see Figure 3, Table 3). These results go hand in hand with the significantly higher BDI-I scores of female PD patients in the general linear mixed model (see Figure 2).

**Table 3:** Fixed effects of the generalized linear mixed model and *post-hoc* repeated measures correlations for the BDI-I scores.

**Source**	***F***	**df1**	**df2**	***p*-values**
DBS-time	0.972	2	50	0.386
Sex	8.720	1	17	0.009
DBS-time*Sex	1.050	2	50	0.357
***Post-hoc***	**r**_**rm**_	**df**	**CI**	***p*****-values**
BDI-I vs. H&Y, women	−0.234	9	−0.71 0.248	0.488
BDI-I vs. H&Y, men	0.322	19	−0.12 0.739	0.154
BDI-I vs. LEDD, women	0.621	15	−0.12 0.878	0.008
BDI-I vs. LEDD, men	0.220	21	−0.13 0.719	0.314

*The reported values for the fixed effects are F-values, degrees of freedom (df1 and df2), and p-values. The reported values for the post-hoc repeated measures correlations are rmcorr-values (r_rm_), degrees of freedom (df), confidence intervals (CI) and p-values*.

### The Parkinson's Disease Questionnaire (PDQ-39)

**Figure 4:**
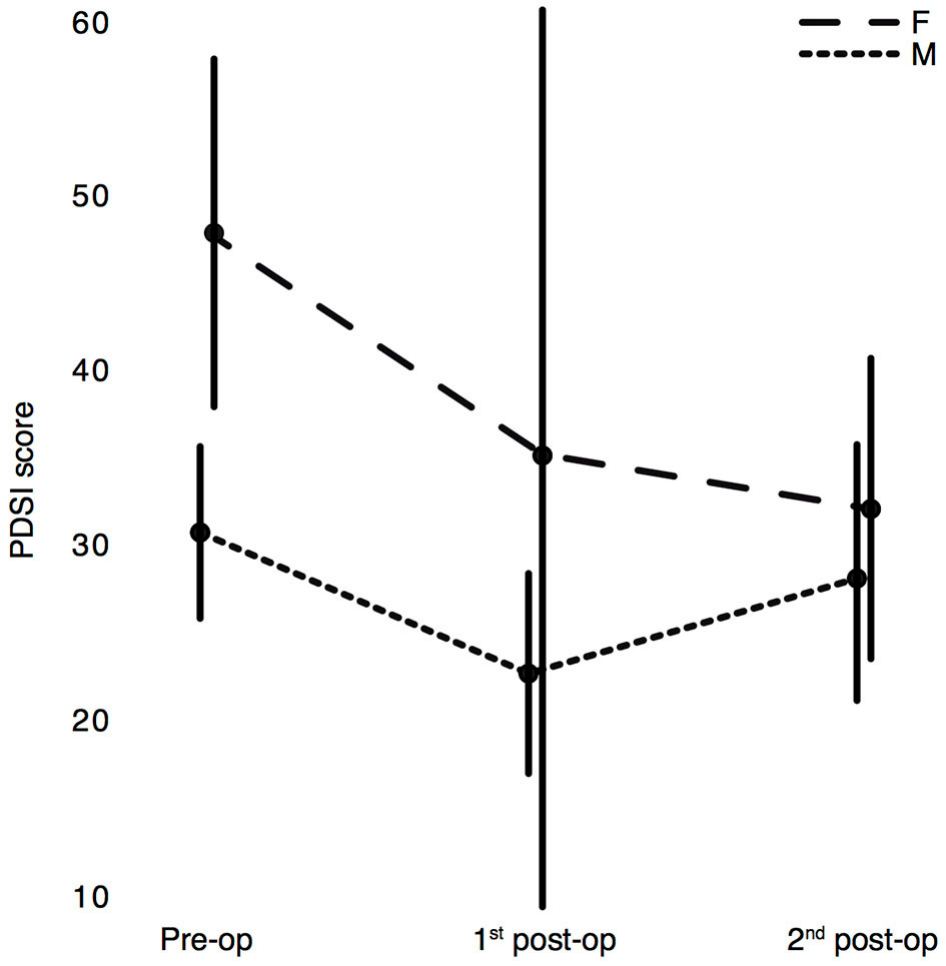
Estimated marginal means of the Parkinson's Disease Questionnaire (PDQ-39) sum score PDSI with 95% CI. Assessment of PDSI scores in PD patients at the three experimental stages: pre-operation, 1st post-operation, 2nd post-operation separated by sex: F = female, M = male. Higher scores correspond to a worse QoL.

The generalized linear mixed model revealed that the PDSI scores were significantly modulated by the factors DBS time and sex as well as by the interactions between factors: DBS time ^*^ sex, DBS time ^*^ H&Y pre-op, and DBS time ^*^ LEDD pre-op (Table 4). In particular, the QoL of PD patients was ameliorated significantly after surgery (pre-operative vs. 1st post-operative: *p* = 0.005, Sidak corrected) and remained on a similar level at the 2nd post-operative stage (pre-operative vs. 2nd post-operative: *p* = 0.048; 1st vs. 2nd post-operative: *p* = 0.422, Sidak corrected). In line with the results from the BDI, female patients reported significantly worse PDSI scores than male patients (*p* = 0.046, Sidak corrected), but their PDSI scores converged to a level similar to that of male patients at the 2nd post-operative stage (Figure 4).

**Table 4:** Fixed effects of the generalized linear mixed model and *post-hoc* repeated measures correlations for the PDQ-39 sum score PDSI.

**Source**	***F***	**df1**	**df2**	***p*-values**
DBS-time	6.432	2	6	0.029
Sex	10.727	1	3	0.046
H&Y pre-op	1.593	1	17	0.224
LEDD pre-op	5.628	1	1	0.333
DBS-time * Sex	5.386	2	12	0.022
DBS-time * H&Y pre-op	5.793	2	10	0.021
DBS-time * LEDD pre-op	6.266	2	6	0.034
***Post-hoc***	**r**_**rm**_	**df**	**CI**	***p*****-values**
PDSI vs. H&Y, women	0.201	9	−0.53 0.763	0.554
PDSI vs. H&Y, men	0.390	17	−0.44 0.699	0.098
PDSI vs. LEDD, women	0.251	13	0.020 0.663	0.366
PDSI vs. LEDD, men	0.257	19	−0.04 0.630	0.260

*The reported values for the fixed effects are F-values, degrees of freedom (df1 and df2), and p-values. The reported values for the post-hoc repeated measures correlations are rmcorr-values (r_rm_), degrees of freedom (df), confidence intervals (CI) and p-values*.

**Figure 5:**
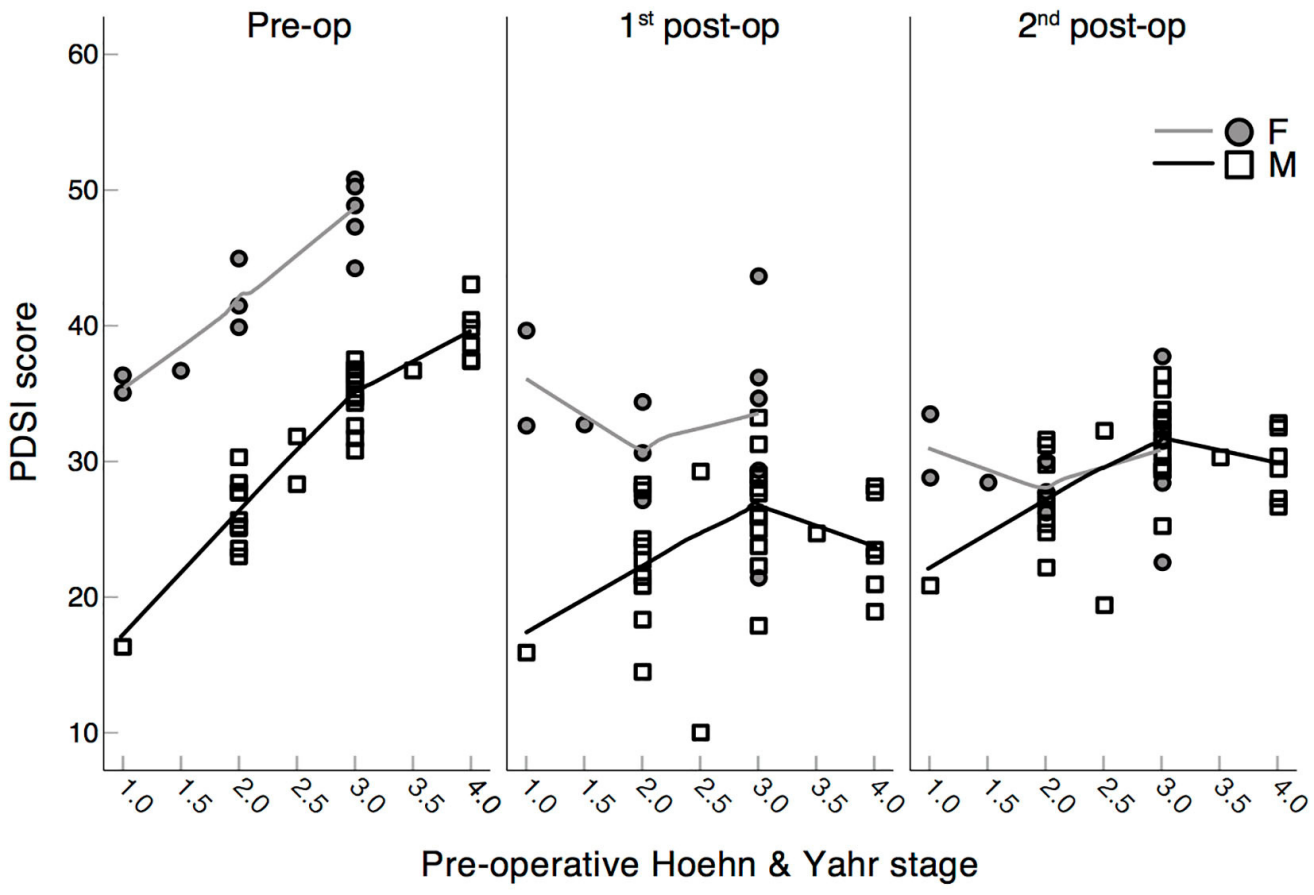
Scatter plots of PDSI scores (PDQ-39) and pre-operative Hoehn & Yahr stage. Assessment of scores in PD patients at the three experimental stages: pre-operation, 1st post-operation, 2nd post-operation separated by sex: F = female, M = male. Higher PDSI scores correspond to a worse QoL. Lines represent Loess fit lines with 99% proportion of data points and Epanechnicov's kernel function.

**Figure 6:**
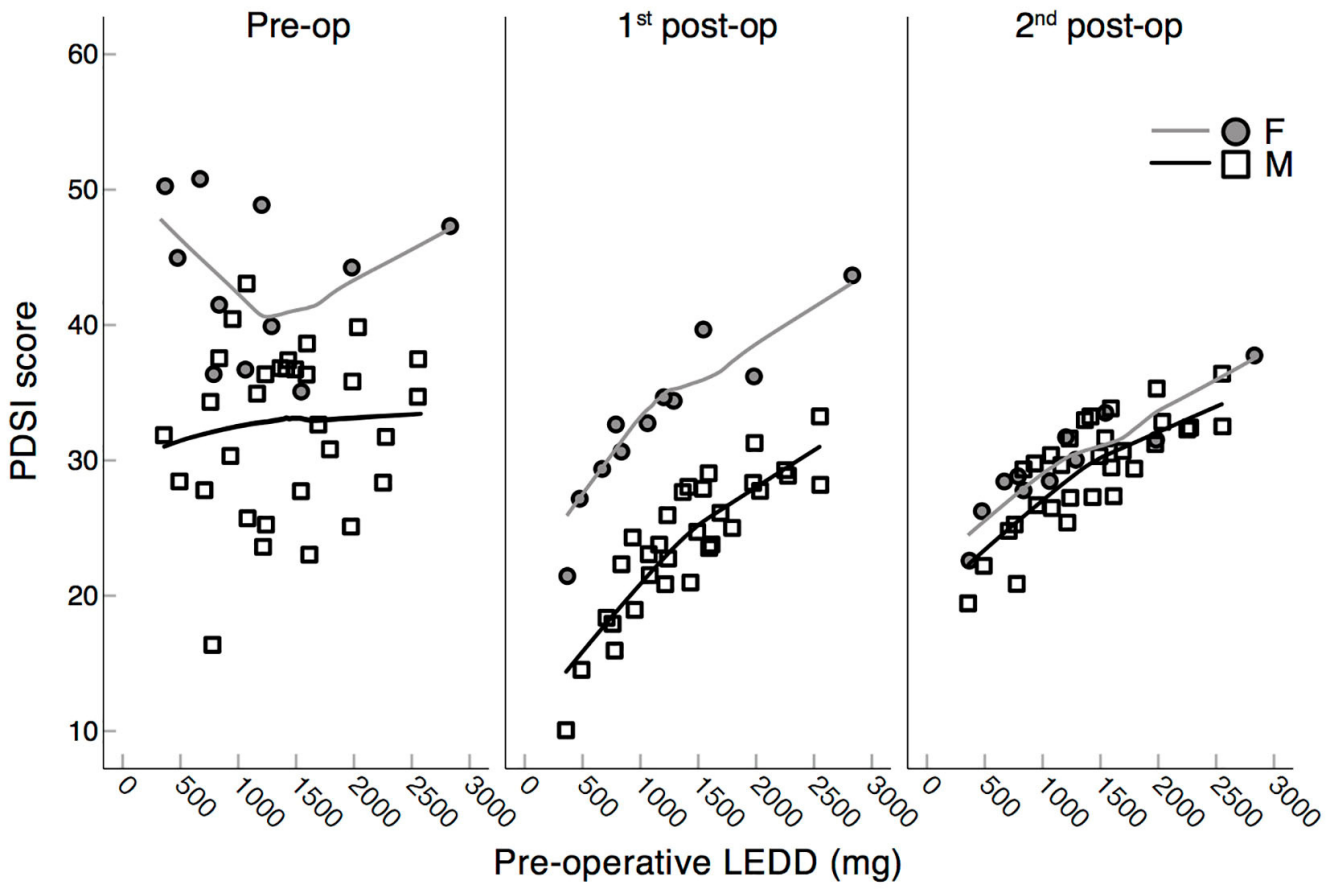
Scatter plots of PDSI scores (PDQ-39) and pre-operative LEDDs. Assessment of scores in PD patients at the three experimental stages: pre-operation, 1st post-operation, 2nd post-operation separated by sex: F = female, M = male. Higher PDSI scores correspond to a worse QoL. Lines represent Loess fit lines with 99% proportion of data points and Epanechnicov's kernel function.

Importantly, the significant interactions between the factors DBS time ^*^ H&Y pre-op, and DBS time ^*^ LEDD pre-op revealed by the generalized linear mixed model for the PDSI scores (Table 4) means that higher *pre-operative* H&Y and LEDD scores may predict worse QoL scores at the three planned stages (see details in Figures 5, 6). Pre-operatively, higher H&Y scores clearly correlated with worse QoL scores, while post-operatively this correspondence was disrupted (see Figure 5). In fact, *post-hoc* repeated measures correlations did not show any significant correspondence between the H&Y and PDSI scores at the three planned stages, either for female or for male patients (see Table 4): *pre-operative* H&Y scores reliably predict worse QoL scores only at the preoperative stage (see pre-op panel in Figure 5).

In the case of PDSI scores and *pre-operative* LEDDs, higher *pre-operative* LEDDs seemed to predict worse *post-operative* QoL scores (*p* = 0.034; see Figure 6).

### Linear Regression Between Follow-Up Time and BDI-I and PDSI Scores

Since disease progression may play a major role in depression and perceived QoL, a fine-graded post-hoc analysis of the factor “follow-up time” was performed. A linear regression was calculated correlating the months elapsed between the 1st and the 2nd post-operation surveys with the differences between the BDI and PDSI scores reported at these two stages. There were slightly positive, but not significant regression coefficients for both BDI and PDSI scores (BDI-I: 0.311, [*F*_1, 20_ = 3.467, *p* = 0.077], with an *R*^2^ of 0.148. PDSI: 0.492, [*F*_1, 21_ = 3.403, *p* = 0.079], with an *R*^2^ of 0.139). In summary, only about 14% of the variance in the post-operative BDI-I and PDSI scores could be explained by the factor follow-up time elapsed between the two post-operative stages.

### Clinical and Motor Scores

Pre-operatively, the median motor score of the Unified Parkinson's Disease Rating Scale (UPDRS-III) (67) was 36 ± 18 IQR after overnight withdrawal of anti-parkinsonian medication, while after intake of levodopa it was reduced to 16 ± 13 IQR (*p* < 0.0001; Wilcoxon signed rank test). This resulted in a median symptom improvement of 53%. The preoperative comparisons between the UPDRS-III scores of male and female patients revealed that women had a median symptom improvement of 62% and men of 50%; these improvements were not significantly different (*p* = 0.063; Wilcoxon rank sum tests). At the two postoperative stages, the UPDRS-III scores of male and female patients, undergoing their standard therapy regime, did not differ significantly (*p* ≥ 0.6 for both comparisons; Wilcoxon rank sum tests). The median pre-operative levodopa equivalent daily dose (LEDD) in mg was 1,263 ± 861 IQR [conversion factors used for the calculation of LEDD from (68)]. During the first period of post-operative questionnaires, LEDD was reduced to 767 mg ± 519 IQR (pre-operative vs. 1st post-operative: *p* < 0.0001; Wilcoxon signed rank test). During the second period of post-operative questionnaires, the median LEDD was 1,020 mg ± 679 IQR (pre-operative vs. 2nd post-operative: *p* = 0.0003; Wilcoxon signed rank test). Paired Wilcoxon signed rank tests between the scores of the two post-operative stages revealed a significant increase in LEDD at the 2nd post-operative stage (*p* = 0.001). Comparisons between the LEDDs of male and female patients at the three stages did not reveal any significant differences (*p* ≥ 0.1 for all three comparisons; Wilcoxon rank sum tests). Further clinical and motor scores are reported in Table 1.

### Stereotactic Reconstruction of Active Electrode Contacts

**Figure 7:**
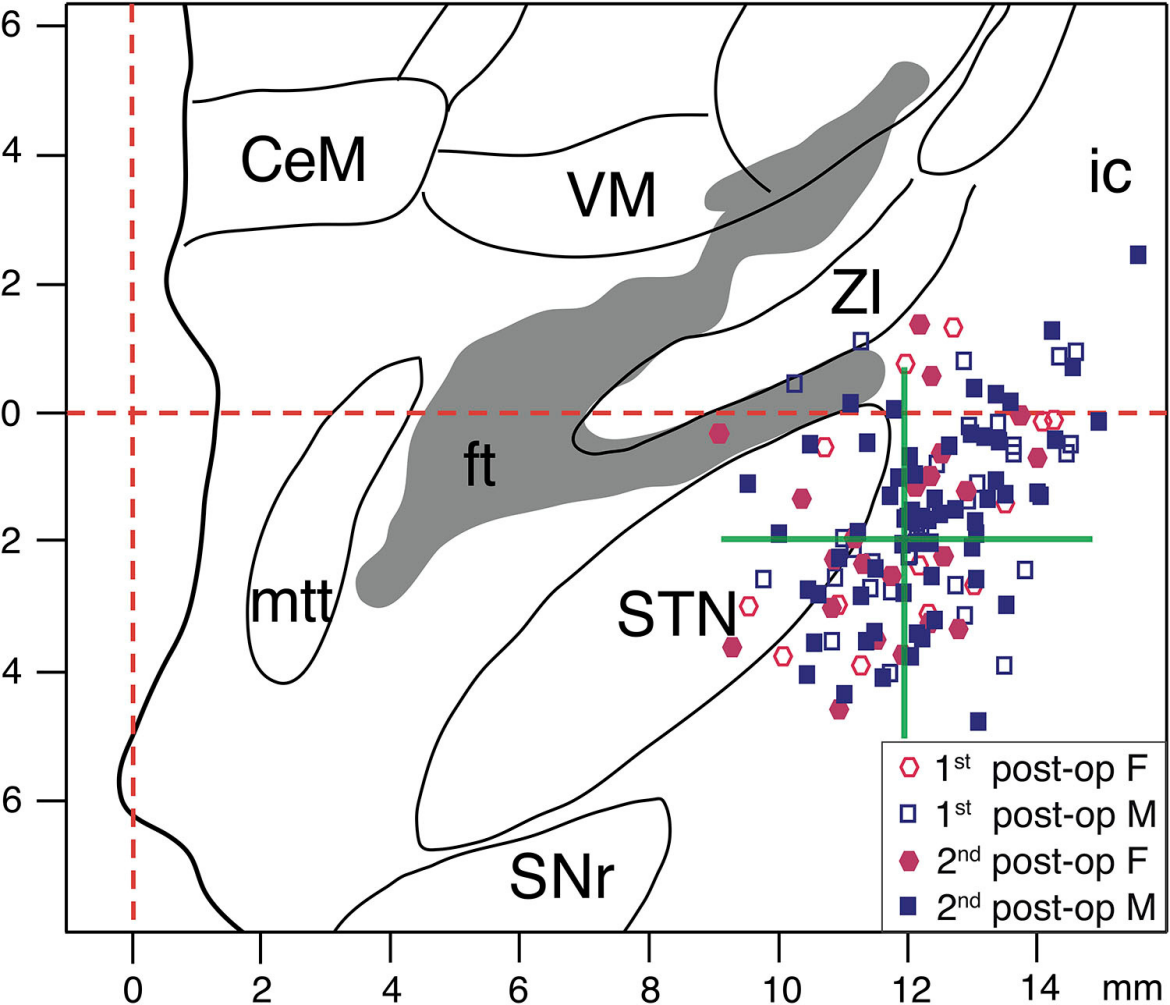
Stereotactic reconstruction of active DBS contacts. The active DBS contacts for both hemispheres are superimposed on a frontal section of the stereotactic atlas of Morel et al., at mid-commissural point level (69). Empty and filled circles represent the active contacts for female patients at the 1st and 2nd post-operative stages, respectively. Empty and filled squares represent the active contacts for male patients at the 1st and 2nd post-operative stages, respectively. The interrupted red lines indicate midline and AC-PC level. The green cross represents the averaged stereotactic target coordinates aimed for the study group. In patients with two adjacent active contacts (cathodes), the averaged x, y, z stereotactic coordinates of those pairs have been plotted. CeM, centralmedialnucleus; ft, fasciculus thalamicus; ic, internal capsule; mmt, mammillothalamic tract; SNr, substantia nigra, pars reticulata; STN, subthalamic nucleus; VM, ventral medial nucleus; ZI, zona incerta.

For all patients, final electrode placement was guided by the results of the intraoperative MER and test stimulation. In most hemispheres, either the central (67/92, 73%) or the anterior trajectory (17/92, 18%) was chosen for the permanent implantation of the DBS electrodes. In seven hemispheres, the electrodes were implanted in the medial trajectory (8%), and only in one case the posterior trajectory was chosen (1%). For the whole group, at the 1st post-operative stage, the stereotactic coordinates of the active contacts relative to the mid-commissural point (MCP; mean ± SD in mm) were x = 12.2 ± 1.2, y = 0.2 ± 1.6, z = 1.6 ± 1.5, while at the 2nd post-operative stage they were x = 12.2 ± 1.3, y = 0.1 ± 1.5, z = 1.7 ± 1.5 (x = lateral to midline, y = anterior to MCP, z = inferior to AC-PC level, values for both hemispheres). For female patients at the 1st post-operative stage, the stereotactic coordinates of the active contacts relative to the MCP were x = 12.0 ± 1.3, y = 0.4 ± 1.7, z = 1.5 ± 1.6, while at the 2nd post-operative stage they were x = 11.8 ± 1.2, y = 0.1 ± 1.6, z = 1.8 ± 1.5. The reconstructed stereotactic coordinates of the active contacts for male patients at the 1st post-operative stage were x = 12.3 ± 1.2, y = 0.1 ± 1.6, z = 1.6 ± 1.4, and at the 2nd post-operative stage they were x = 12.3 ± 1.2, y = 0.1 ± 1.5, z = 1.6 ± 1.5. The stereotactic coordinates of the active contacts of male and female patients did not differ significantly either at the 1st or at the 2nd post-operative stage (*p* ≥ 0.1 for all comparisons; Wilcoxon rank sum tests). In Figure 7, the localizations of the reconstructed active DBS contacts for both right and left electrodes are superimposed on a frontal section of the stereotactic atlas of Morel et al. (69).

## Discussion

In this evaluation of mid- and long-term effects of STN-DBS on personality traits of PD patients, no detrimental alterations of personality were reported in the study group. STN-DBS seemed to exert sex-dependent effects on mood and QoL in PD patients: women profited more than men from the STN-DBS treatment in terms of lowering of depression scores and amelioration of QoL. Crucially, *pre-operative* higher LEDD scores predicted worse *post-operative* QoL scores.

### FPI-R

The main goal of our study was to track possible changes in personality following STN-DBS surgery, by means of a self-administered survey (25, 70). In our patient group, the personality traits considered did not seem to change substantially after surgery. Although, some studies have shown specific sex-dependent effects on the outcomes of STN-DBS, separating the patients by sex did not reveal a significant modulation in personality traits in our patients (29, 30). This could be ascribed to a lack of sensitivity of the FPI-R to these dimensions in the phases chosen for the study or to the non-compliance of the patients reporting their psychic and personal state to the therapists. It should be noted that the FPI-R has not been validated for PD, but at present a test specifically designed to track possible personality changes in Parkinson patients is not available. Another explanation could be a lack of introspection when completing self-administered questionnaires. To address this issue, future studies should consider structured face-to-face interviews with the patients and with the caregivers, alternative instruments such as the Minnesota Multiphasic Personality Inventory, and questionnaires specifically aimed at the caregivers (21, 44, 71–73). All things considered, there are still good reasons to assume that the considered personality traits remain stable after long-term treatment with STN-DBS. We would like to draw particular attention to the significant decrease in “Life Satisfaction” reported at the 1st post-operative stage in comparison to the pre-operative level, as evidenced by the planned *post-hoc* pairwise contrasts. Some patients could have been disillusioned about the impact of the DBS treatment on their life and may have rated their life satisfaction as slightly lower than before the surgery, while 1 year later this judgment was mitigated and returned to pre-operative level again. It is known that after neurosurgery for STN-DBS, patients tend to overestimate their pre-operative functioning, hence misperceiving their post-operative improvements (74). Moreover, it is possible that unrealistic pre-operative expectations worsen patients' satisfaction with the STN-DBS results as well (75, 76). It is, therefore, conceivable that after reporting a moderate satisfaction with life shortly after surgery, this judgement was revisited and increased to a more satisfying level in the long term. Also evidenced by the planned *post-hoc* contrasts, a reduced performance orientation was found at the 2nd post-operative stage in comparison to the pre-operative stage. This late reduction in performance orientation could be associated with the patients' satisfaction with their status quo, but also with the apathetic state some patients may show after a strong post-operative reduction of dopaminergic medication (64, 77). Unfortunately, a specific assessment of apathy level was not performed in this trial. In future surveys on personality traits, apathy changes should be tracked in PD patients undergoing surgery for STN-DBS, to correlate them with performance orientation.

### BDI-I

On average, no signs of depression were evidenced by the BDI-I scores in our cohort, which remained substantially unchanged throughout all the three considered stages. This aspect is particularly relevant, since depressive symptoms have a great negative impact upon QoL and personality (9, 78). In general, women showed more depressive symptoms than men, in line with results from other groups (27, 79, 80). Repeated measures correlations confirmed a direct link between the level of depressive signs in female patients and their individual LEDD at the three stages. The close relationship between levodopa treatment, the serotonergic system and depressive symptoms has been previously described: The higher the LEDD, the stronger the fluctuations patients may experience, and the greater the frontal lobe dysfunctions involving the dopaminergic, serotonergic and noradrenergic systems may be, mediating depressive symptoms (81–84). It remains to be clarified why the correlation between LEDDs and BDI scores was significant only for female patients. The differences between BDI-I scores reported by men and by women may be due to sex-biased items and therefore result in higher depression scores in female than in male patients (85): male patients may have played down some signs of depression and therefore masked the correlation to the LEDD.

### PDQ-39

The sustained mid- and long-term improvement in the global health status of the patients of our cohort is in line with improvements reported in previous studies (4, 6, 7, 86). Our results indicated that female patients declared a poorer quality of life than male patients pre-operatively (79). But after surgery, women's quality of life improved more consistently than men's, converging to similar levels at the 2nd post-operative stage. It therefore seems as though women benefit more from STN-DBS surgery in terms of QoL than their male counterpart do (28). Importantly, in both men and women, higher *pre-operative* LEDD scores predicted worse *post-operative* QoL scores (see 1st and 2nd post-op panels in Figure 6). If confirmed by subsequent studies, this aspect may speak in favor of an earlier neurostimulation approach; i.e., eligible patients could be operated before the limits of dopamine substitution therapy are reached (87, 88).

### Limitations of the Approach

Missing data and heterogeneous sample sizes are some of the most troublesome issues in longitudinal biomedical studies. An overall response rate of more than 76% was obtained, which is in line with the prevalent values of self-administered surveys in health care (89, 90). The explanations for not completing the questionnaires entirely or returning them on time were diverse but were mainly associated with forgetfulness and burden. To deal with missing data and unequal sample sizes, a mixed model approach was chosen (56): mixed models can handle incomplete data sets and unbalanced groups, and parameters can be estimated successfully with the available data (57). Nevertheless, it remains to be clarified whether patients satisfied with the outcomes of the DBS therapy were possibly more keen to complete the questionnaires, while patients discontent with the DBS results were more reluctant to report their issues to the therapeutic team (71). Another confounding factor is the non-anonymized questionnaires: patients may have been less candid with their answers to avoid giving unpleasant feedback to the therapeutic team, so that more positive and neutral answers were given.

Other serious confounding factors are the locations of the active lead contacts and the DBS programming skills of clinic personnel. In fact, depending on active contact location and the stimulated structures, there can be serious adverse neuropsychiatric consequences that can only be partially compensated by skilled therapists (73, 91). At our institution, a task force of a few neurologists and clinical personnel is specifically trained for and dedicated to DBS therapy. Contacts located too far ventromedially or laterally are avoided to lower the likelihood of neuropsychiatric symptoms or accidental stimulation of internal capsule fiber tracks, respectively (64, 92, 93). In the study group considered, the reconstructed location of active contacts was consistent with the planned and reported stereotactic coordinates for STN-DBS [(94, 95); see Figure 7]. Furthermore, the localization of the lead contacts did not differ significantly between female and male patients at the two post-operative stages; thus it should not account for the differences in outcomes reported by women and men.

The small number of women participating in the study reflects the uneven distribution of women and men undergoing neurosurgery for STN-DBS worldwide (39, 40), but it also limited the strength of the conclusions we could draw from our data. A higher number of participants, and therefore more women, should be aimed for in a larger, multi-center trial.

Finally, the wide time ranges, in particular the ones considered for the 2nd post-operative stage, may be an issue, since disease progression may have played a role in the reported depression and QoL scores. In our study, the elapsed time between the two post-operative time ranges did not significantly affect the reported mood or QoL. However, in future trials, narrower time ranges should be used for administering the questionnaires in the follow-up periods.

### Conclusions

Stable personality traits were observed in the mid- and long-term after surgery for STN-DBS in our cohort of PD patients. The sustained improvement in QoL after surgery was particularly advantageous for female patients, for whom an interchange between LEDD and the severity of depressive symptoms was also found. Importantly, higher *pre-operative* LEDD scores were associated with worse *post-operative* QoL scores. This aspect could speak in favor of an early DBS approach, before severe levodopa-induced sequelae appear (87, 88). These results may further encourage the treatment of PD patients by chronic STN stimulation at early stages of the disease and could reduce concerns about possible detrimental effects of the STN-DBS therapy on personality traits and mood, but need to be confirmed by a large, multi-center study. In the future, standardized assessment of personality should be evaluated in the daily therapeutic routine, in order to gain a more comprehensive picture of patients' psychological condition throughout the decade-lasting STN-DBS therapy (21, 71). In the selection of possible candidates for STN-DBS treatment, it may be beneficial to focus more on the sex-related effects STN-DBS seems to exert on male and female PD patients, since female patients seem to profit more from STN-DBS in terms of lowering depressive symptoms and increasing QoL than male patients. This approach may help to overcome the sex disparities among PD patients treated with DBS, since women are still strongly under-represented (39, 96).

## Data Availability Statement

The datasets generated for this study are available on request to the corresponding author.

## Ethics Statement

The studies involving human participants were reviewed and approved by Ethik-Kommission der Ärztekammer Hamburg. The patients/participants provided their written informed consent to participate in this study. Written informed consent was obtained from the individual(s) for the publication of any potentially identifiable images or data included in this article.

## Author Contributions

The work presented here was carried out in collaboration between all authors. CM, AG, and WH: conception of research project and designed methods and assessments. AG, AD, CM, CB, MP-N, CG, MW, AE, WH, JK, MS, CO, MO, and KM: organization of research project. AG, AD, WH, MP-N, CB, CM, CO, MO, and KM: execution of research project. JK, AD, WH, HP, and AG: designed the statistical analysis. AD, JK, HP, and AG: execution of statistical analysis and analyzed the data. AG, AD, JK, CM, and WH: reviewed and critique of statistical analysis, discussed analyses, interpretation, and presentation. AD and AG: writing of the first draft of the manuscript. AG, CM, WH, JK, MS, MP-N, CB, CG, MW, AE, CO, MO, KM, and HP: reviewed and critique of the manuscript. All authors have contributed to, seen and approved the manuscript.

## Conflict of Interest

AG, CM, WH, and CB have occasionally been reimbursed for travel expenses from Medtronic Inc. CO, MO, and KM have been funded for workshops by Medtronic Inc. and Abbott. CB received compensation for lectures from Abbvie, Bial, TAD Pharma, UCB Pharma and Zambon. CG reports personal fees and other from Bayer Healthcare and Boehringer Ingelheim, personal fees from Abbott, Acticor Biotech, Amgen, BMS, Sanofi Aventis, and Prediction Biosciences. CM served as medico- scientific consultant to Abbott. WH received lecture fees and honoraria for serving on advisory boards and travel grants from Boston Scientific, Medtronic, and Abbott. MP-N received lecture fees from Abbott and Licher, and served as consultant for Medtronic, Boston scientific and Abbvie. The remaining authors declare that the research was conducted in the absence of any commercial or financial relationships that could be construed as a potential conflict of interest.
